# Comparison of valvuloplasty and replacement for surgical treatment of tricuspid infective endocarditis

**DOI:** 10.1186/s12872-023-03248-1

**Published:** 2023-04-28

**Authors:** Linfeng Xie, Xiaodong Chen, Jian He, Sixian Lin, Xingfeng Chen, Qingsong Wu, Ling Chen, Jingxiang Zhuang, Zhihuang Qiu, Liangwan Chen

**Affiliations:** 1 Department of Cardiovascular Surgery, Union Hospital, Fujian Medical University, Fuzhou, Fujian 350001 P. R. China; 2 Fujian Medical University, Fuzhou, Fujian 350001 P. R. China; 3 Fujian Key Laboratory of Cardio-Thoracic Surgery, Fujian Medical University, Fuzhou, Fujian 350001 P. R. China; 4 Fujian Provincial Center for Cardiovascular Medicine, Fuzhou, Fujian 350001 P. R. China; 5 Department of Emergency, Nanjing County Hospital, Zhangzhou, Fujian P. R. China

**Keywords:** Tricuspid infective endocarditis, Surgical treatment, Tricuspid valve replacement, Tricuspid valvuloplasty

## Abstract

**Background:**

Owing to the increase in both intravenous drug injections and intracardiac and vascular interventional treatments among drug users, the incidence of infective endocarditis (IE) involving the tricuspid valve, which sits between the two right heart chambers, has gradually increased. This study aimed to compare the clinical outcomes of different surgical procedures for tricuspid infective endocarditis (TIE).

**Methods:**

We retrospectively analyzed fifty-six patients who underwent tricuspid valve surgery at our hospital from January 2006 to August 2019. All patients were diagnosed with TIE and indicated a need for surgery. Perioperative and follow-up data were collected to summarize and analyze the clinical outcomes of different surgical approaches, including tricuspid valvuloplasty (TVP) and tricuspid valve replacement (TVR) for TIE.

**Results:**

Cardiopulmonary bypass (CPB) time, aortic cross-clamp (ACC) time, postoperative mechanical ventilation time, and intensive care unit (ICU) stay time were shorter in the TVP group than in the TVR group. Additionally, the incidence of red blood cell transfusion and postoperative complications was lower in the TVP group than in the TVR group. The postoperative 30-day mortality rates were similar between both the groups. Fifty-two patients were followed up for an average of 5.50 ± 3.79 years. The postoperative 3-, 5-, and 7-year survival rates were 100%, 100%, and 95.5% in the TVP group and 96.7%, 96.7%, and 96.7% in the TVR group, respectively. The 5-year and 10-year reoperation rates were 0% and 0% in the TVP group and 6.7% and 20% in the TVR group, respectively.

**Conclusion:**

Both TVR and TVP for TIE significantly improved the functional status of the heart with satisfactory efficacy. TVP was found to be superior to TVR in reducing the need for postoperative blood transfusions, reducing the risk of postoperative complications, and reducing the need for long-term reoperations.

## Background

Right-sided infective endocarditis (IE) accounts for 5–10% of all IE cases. Approximately 90% of right-sided IE cases involve the tricuspid valve. This diagnosis is known as tricuspid infective endocarditis (TIE) [1, 2]. In recent years, owing to the increase in both intravenous drug injections and intracardiac and vascular interventional treatments among drug users, the incidence of IE involving the tricuspid valve within the right heart chambers has gradually increased [3, 4].

Surgical treatment for TIE includes radical removal of the infected tissue, recovery of valve physiological function, and improvement in cardiac function. There are two types of routinely performed surgery: tricuspid valvuloplasty (TVP) and tricuspid valve replacement (TVR) [5, 6]. TVP mainly includes Kay suture valvuloplasty, De Vega annuloplasty, pericardial patch valvuloplasty and prosthetic ring annuloplasty. Different TVP procedures are suitable for the treatment of TIE depending on the cause of tricuspid valve regurgitation [7, 8]. TVR includes tricuspid biological valve replacement and tricuspid mechanical valve replacement. TVR should be considered when tricuspid valve disease is severe or in cases where tricuspid valvuloplasty fails [9, 10]. Because of the low incidence of TIE, some patients can be cured with drug therapy. Relatively few patients require surgical treatment for TIE. Few comparative studies have reported on the different surgical methods for TIE. Therefore, this study aimed to compare the clinical early- and mid-term outcomes of different surgical procedures, i.e., TVP and TVR, for TIE.

## Methods

### Patients

In this retrospective study, we examined fifty-six patients with TIE who were surgically treated at our institution from January 2006 to August 2019. These patients were divided into the TVP group (n = 23) and the TVR group (n = 33). All patients were diagnosed according to the modified Duke criteria, or TIE was confirmed by postoperative pathology. Preoperative transthoracic echocardiography showed tricuspid valve vegetation in all cases [11].

After admission, further laboratory tests were performed, and symptomatic treatment was administered. Most patients in the study had a previous TIE diagnosis and had been transferred to our facility from lower-level hospitals. A standard blood culture was performed when TIE was suspected, and the corresponding antibiotic treatment was administered. Routine anti-infective treatment was administered for 2 weeks post-surgery.

### Indications for surgery

The indications for surgery were: (1) septicemia that cannot be controlled by drugs; (2) right ventricular insufficiency that cannot be controlled by drugs; (3) formation of perivalvular abscess; (4) right ventricular IE caused by fungi or other refractory pathogens; (5) recurrent pulmonary embolism; (6) combined right- and left-sided IE; (7) persistent fever; and (8) vegetation diameter > 20 mm.

### Surgical procedure

All operations were performed under general anesthesia. The sternum was cut in the middle, and a cardiopulmonary bypass was established through the ascending aorta and the superior and inferior vena cava. After cardiopulmonary bypass, the temperature was uniformly cooled to 32℃, and HTK solution was infused from the root of the aorta to protect the myocardium. The size of the tricuspid annulus, the lesion of the tricuspid valve, the location and size of vegetation, the degree of valve lesion, the destruction of the subvalvular structure, and the formation of perivalvular abscess were examined routinely before operation. Based on the exploration results, different tricuspid valve operations were selected [12, 13]. Vegetation was removed in all cases. In patients with left heart valve disease or other intracardiac malformations that required surgical treatment, the left heart valve surgery or intracardiac malformation correction was performed first, followed by the tricuspid valve surgery. After tricuspid valve treatment, opening and closing of the tricuspid valve, conduction block, and other arrhythmias were monitored. Residual tricuspid regurgitation was observed by drawing water. Post-surgery, the effects of the operation on tricuspid valve activity and residual regurgitation were re-evaluated using transesophageal ultrasonography.

### Tricuspid valvuloplasty

TVP was performed in patients with moderate or severe tricuspid regurgitation; no abnormalities, including calcification, thickening, curl, and shortening, on the tricuspid valve leaflets and subvalvular structures; and a diastolic tricuspid annulus diameter of > 40 mm. TVP includes Kay suture valvuloplasty, De Vega annuloplasty, pericardial patch valvuloplasty, and prosthetic ring annuloplasty.

### Tricuspid valve replacement

TVR was performed when the effect of TVP was unsatisfactory or when severe tricuspid valve insufficiency prevented valvuloplasty during the operation [13, 14].

### End points

The study endpoints were postoperative all-cause mortality and IE recurrence, including relapse and reinfection. The diagnoses of IE relapse and IE reinfection were based on the modified Duke criteria. Relapse was defined as IE caused by any microbial infection within 6 months post-surgery. Reinfection was defined as an infection that was caused by a different microorganism from the primary infection or an infection that occurred more than 6 months after the initial infection [15].

### Statistical analysis

We used SPSS version 23.0 for Windows software for all statistical analyses. Continuous variables with a normal distribution are expressed as mean ± standard deviation (SD) and compared using a student t-test; otherwise, they are expressed as median (Q25, Q75) and compared with a Mann–Whitney U test. Categorical data were shown as number (%) and analyzed using the chi-square test or Fisher’s exact test as appropriate. P value < 0.05 was considered statistically significant. The survival rate and freedom from reoperation rate were calculated by using the Kaplan-Meier method and and Log-Rank test was used to test whether there was any difference between groups.

## Results

### Comparison of preoperative condition between the two groups

No significant differences were noted in age, sex, course of disease, preoperative cardiac function, preoperative complications, or underlying diseases between the two groups (*p >* 0.05). In the TVP group, three patients (13.04%) had prior cardiac surgery, four patients (17.39%) had cardiac pacemaker implantation, four patients (17.39%) had left ventricular valvular disease, and four patients (17.39%) had atrial fibrillation. In the TVR group, three patients (9.09%) had prior cardiac surgery, four patients (12.12%) had cardiac pacemaker implantation, ten patients (30.30%) had left ventricular valvular disease, and six patients (18.18%) had atrial fibrillation. All patients exhibited signs of acute infection such as high fever and leukocytosis. Overall, eight patients (14.28%) had blood culture findings of *Staphylococcus aureus*, three patients (5.36%) had findings of *Staphylococcus epidermidis*, three patients (5.36%) had findings of *Enterococcus faecalis*, 19 patients (33.93%) had findings of *Streptococcus bovis*, and three patients (5.36%) had findings of *Acinetobacter baumannii*. In the remaining patients, no bacterial growth was detected, most likely due to previous antibiotic therapy.

In the TVP group, the vegetation diameter was longer than 20 mm in six patients (26.09%) and longer than 30 mm in one patient (4.35%). Moderate or severe tricuspid regurgitation was noted in twenty patients (86.96%) in the TVP group. In the TVR group, the vegetation diameter was longer than 20 mm in twelve patients (36.36%) and longer than 30 mm in four patients (12.12%). Moderate or severe tricuspid regurgitation was noted in twenty-eight patients (84.85%) in the TVR group. No significant differences were noted in the above data and clinical indices, such as right atrial diameter, right ventricle diameter, left ventricular ejection fraction, and pulmonary artery pressure between the two groups (*p* > 0.05) (Table 1).

**Table 1:** Preoperative characteristics

Valuables	TVP group n = 23	TVR group n = 33	P-value
Age (year)	37.57 ± 15.05	42.24 ± 15.51	0.266
Male (n, %)	10(43.48%)	17(51.52%)	0.554
Course of disease (day)	48.52 ± 62.08	32.82 ± 24.18	0.192
Hypertension (n, %)	0	2(6.06%)	0.343
Intravenous drug abuse (n, %)	3(13.04%)	4(12.12%)	0.613
Infected pacemaker (n, %)	4(17.39%)	4(12.12%)	0.812
Prior cardiac surgery (n, %)	3(13.04%)	3(9.09%)	0.479
Atrial fibrillation (n, %)	4(17.39%)	6(18.18%)	0.614
Pulmonary embolism (n, %)	2(8.70%)	3(9.09%)	0.670
Abnormal hepatic function (n, %)	1(4.35%)	1(3.03%)	0.666
Associate left heart valvular disease	4(17.39%)	10(30.30%)	0.272
**Cardiac function classification (NYHA)**			0.834
NYHA class II (n, %)	6(26.09%)	10(30.30%)	
NYHA class III (n, %)	15(65.22%)	19(57.58%)	
NYHA class IV (n, %)	2(8.70%)	4(12.12%)	
**Preoperative biochemical data**			
Leucocytes > 10*10^9^/L (n, %)	15(65.22%)	24(72.73%)	0.548
Hemoglobin (g/L)	98.52 ± 13.85	93.00 ± 16.87	0.201
Albumin (g/L)	30.64 ± 4.34	29.35 ± 4.54	0.186
Positive blood culture (n, %)	16(69.57%)	20(60.61%)	0.491
Staphylococcus aureu	3(13.04%)	5(15.15%)	
Staphylococcus epidermidis	2(8.70%)	1(3.03%)	
Streptococcus bovis	9(39.13%)	10(30.30%)	
Acinetobacter baumannii	2(8.70%)	1(3.03%)	
Enterococcus faecalis	0	3(9.09%)	
**Preoperative echocardiography**			
RAD (mm)	51.30 ± 9.46	56.18 ± 11.26	0.095
RVD (mm)	22.10 ± 4.00	24.32 ± 4.45	0.061
Vegetations size (mm)	18.37 ± 5.15	20.35 ± 6.96	0.253
PAP (mmHg)	53.78 ± 17.42	52.09 ± 17.33	0.721
Moderate or severe tricuspid regurgitation (n, %)	20(86.96%)	28(84.85%)	0.572
LVEF (%)	64.36 ± 5.20	66.31 ± 7.15	0.277

Continuous variables were present as mean ± standard deviation (SD). Categorical variables were shown as number (%). The student t test or Man-Whitney U test was used for continuous variables, and Chi-square test used for categorical variables

RAD, right atrial diamete; RVD, right ventricle diameter; PAP, pulmonary artery pressure; LVEF, left ventricular ejection fractions

### Comparison of intraoperative and postoperative data between the two groups

In the TVP group, the CPB time (79.68 ± 19.02 min vs. 107.39 ± 25.64 min, *p* < 0.001), ACC time (50.29 ± 16.14 min vs. 65.52 ± 20.62 min, *p* = 0.005), and mechanical ventilation time (18.65 ± 8.18 h vs. 44.85 ± 57.68 h, *p* = 0.001) were significantly lower than those in the TVR group. The intensive care unit (ICU) stay time (38.13 ± 21.80 h vs. 102.64 ± 142.11 h, *p =* 0.015) and red blood cell transfusion (4.87 ± 3.81U VS 7.55 ± 5.42U, *p =* 0.046) were lower in the TVP group than in the TVR group. However, no significant difference was noted between the two groups in pericardial and mediastinal drainage (590.87 ± 769.86ml vs. 1060.61 ± 1717.50mL, *p* = 0.225) and hospitalization time (27.74 ± 7.82d vs. 29.52 ± 10.55d, *p* = 0.496).

Postoperative complications occurred in two patients in the TVP group and eleven patients in the TVR group. Significant differences were noted in the incidence of postoperative complications between the two groups (8.70% vs. 33.33%, *p* = 0.032). The postoperative complications in the TVP group included one case (4.35%) of myelosuppression and one case (4.35%) of III-degree atrioventricular block. In the TVR group, one patient (3.03%) had hepatic dysfunction, two (6.06%) had acute kidney injury, two (6.06%) had III-degree atrioventricular block, one (3.03%) had low cardiac output syndrome, one (3.03%) had surgical incision infection, two (6.06%) had severe pneumonia, and two (6.06%) had MODS. No perioperative deaths were noted in the TVP group, but one (3.03%) perioperative death was noted in the TVR group **(**Table 2**)**.

**Table 2:** Operative and postoperative data

Valuables	TVP group n = 23	TVR group n = 33	P- value
**Intraoperative time**			
CPB time (min)	79.68 ± 19.02	107.39 ± 25.64	**< 0.001**
ACC time (min)	50.29 ± 16.14	65.52 ± 20.62	**0.005**
**Postperative complication data**			
ICU stay time (h)	38.13 ± 21.80	102.64 ± 142.11	**0.015**
Mechanical ventilation time (h)	18.65 ± 8.18	44.85 ± 57.68	**0.001**
Red blood cell transfusion (U)	4.87 ± 3.81	7.55 ± 5.42	**0.046**
Pericardial and mediastinal drainage (ml)	590.87 ± 769.86	1060.61 ± 1717.50	0.225
Hospitalization time (day)	27.74 ± 7.82	29.52 ± 10.55	0.496
30-day death (n, %)	0	1(3.03%)	0.855
**Complications (total number of cases) (n, %)**	2(8.70%)	11(33.33%)	**0.032**
Acute kidney injury (n, %)	0	2(6.06%)	0.638
Hepatic dysfunction (n, %)	0	1(3.03%)	0.855
Severe pneumonia (n, %)	0	2(6.06%)	0.638
III-degree atrioventricular block (n, %)	1(4.35%)	2(6.06%)	0.747
Low cardiac output syndrome (n, %)	0	1(3.03%)	0.855
Surgical incision infection (n, %)	0	2(6.06%)	0.638
MODS (n, %)	0	1(3.03%)	0.855
Myelosuppression (n, %)	1(4.35%)	0	0.855

Continuous variables were present as mean ± standard deviation (SD). Categorical variables were shown as number (%). The student t test or Man-Whitney U test was used for continuous variables, and Chi-square test used for categorical variables

CPB, cardiopulmonary bypass; ACC, aortic cross-clamp; MODS, multiple organ dysfunction syndrome; ICU, intensive care unit

### Comparison of postoperative follow-up between the two groups

After multi-channel follow-up from 6 months to 14 years, the average follow-up duration was 5.50 ± 3.79 years. Overall, fifty-two patients were followed up successfully: twenty-two patients in the TVP group (95.65%) and thirty in the TVR group (93.75%). In the TVP group, endocarditis relapses was noted in one patient (4.55%), which was cured after antibiotic treatment. Two cases (6.67%) of endocarditis showed reinfections in the TVR group, both of which were cured by tricuspid valve replacement. One patient (4.55%) in the TVP group had heart failure. In the TVR group, one patient (3.33%) had heart failure, and one patient (3.33%) had pulmonary embolism and four patients (13.33%) had artificial valve dysfunction. In the TVP group, one patient (4.55%) died of heart failure 6 years post-surgery, and one patient (3.33%) in the TVR group died of heart failure 2 years post-surgery. The postoperative 3-, 5-, and 7-year survival rates were 100%, 100%, and 95.5% in the TVP group and 96.7%, 96.7%, and 96.7% in the TVR group, respectively. The 5- and 10-year reoperation rates were lower in the TVP group than in the TVR group (Table 3).

**Table 3:** Postoperative follow-up data

Valuables	TVP group n = 22	TVR group n = 30	P -value
**Postoperative recurrence (n, %)**	1(4.55%)	2(6.67%)	0.617
Postoperative relapses (n, %)	1(4.55%)	0	
Postoperative reinfections (n, %)	0	2(6.67%)	
Artificial valve dysfunction (n, %)	0	4(13.33%)	0.101
Pulmonary embolism (n, %)	0	1(3.33%)	0.577
Heart failure (n, %)	1(4.55%)	1(3.33%)	0.672
NYHA class III- IV (n, %)	4(18.18%)	10(33.33%)	0.230
Three-year survival (n, %)	22(100%)	29(96.7%)	1.000
Five-year survival (n, %)	22(100%)	29(96.7%)	1.000
Seven-year survival (n, %)	21(95.5%)	29(96.7%)	1.000
Five-year reoperation (n, %)	0	2(6.67%)	0.502
Ten-year reoperation (n, %)	0	6(20%)	**0.029**
Tricuspid regurgitation above moderate (n, %)	6(27.27%)	0	**0.004**
LVEF (%)	65.18 ± 4.64	62.13 ± 7.30	0.092

Continuous variables were present as mean ± standard deviation (SD). Categorical variables were shown as number (%). The student t test or Man-Whitney U test was used for continuous variables, and Chi-square test used for categorical variables

LVEF, left ventricular ejection fractions

**Fig. 1:**
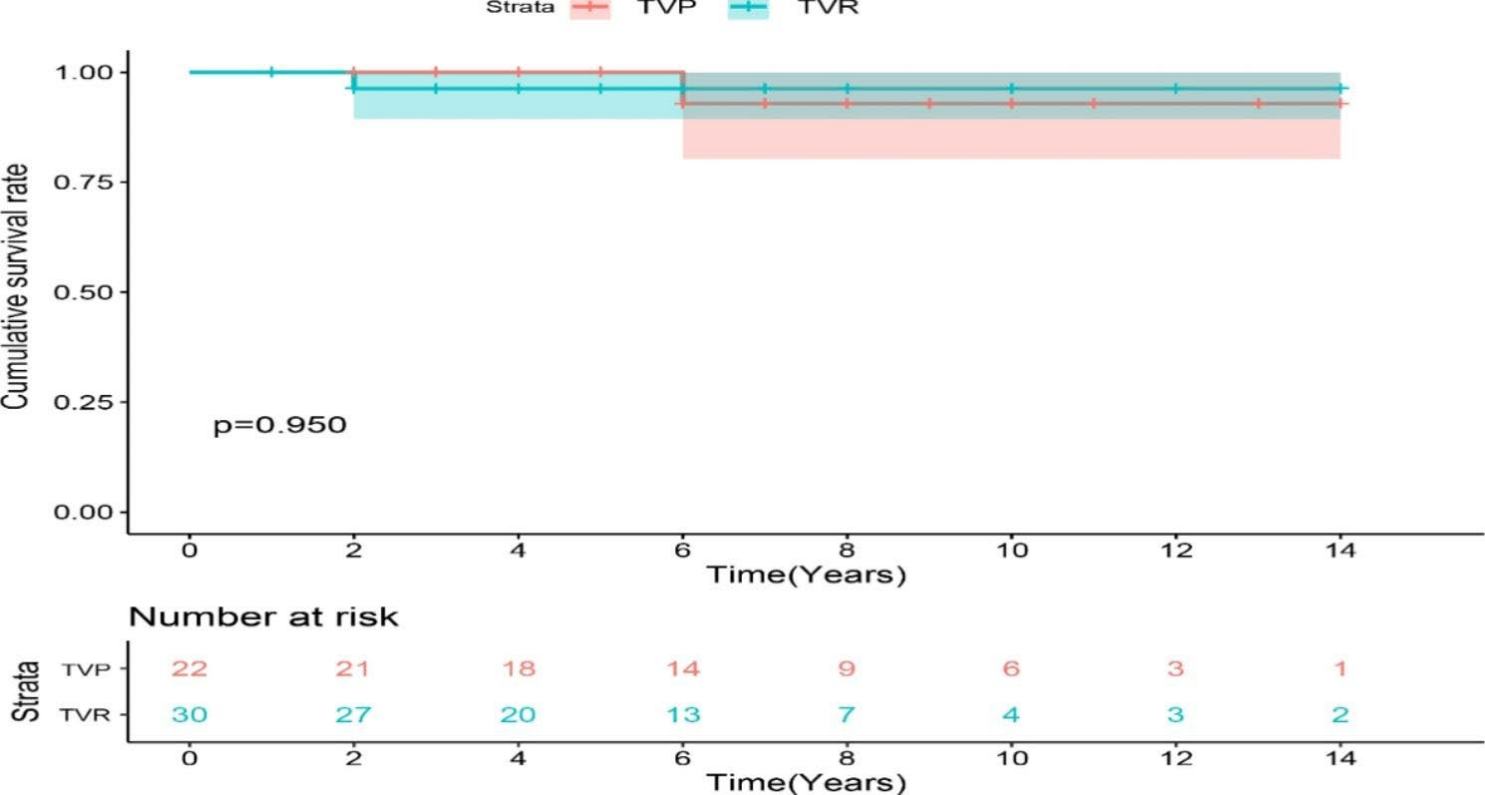
Estimates of survival of patients with TIE who underwent TVP and TVR for surgical treatment

The cumulative survival curve with postoperative death as the end point showed no significant difference in early- and medium-term survival rates between the two groups (log-rank *p* = 0.950) (Fig. 1).

**Fig. 2:**
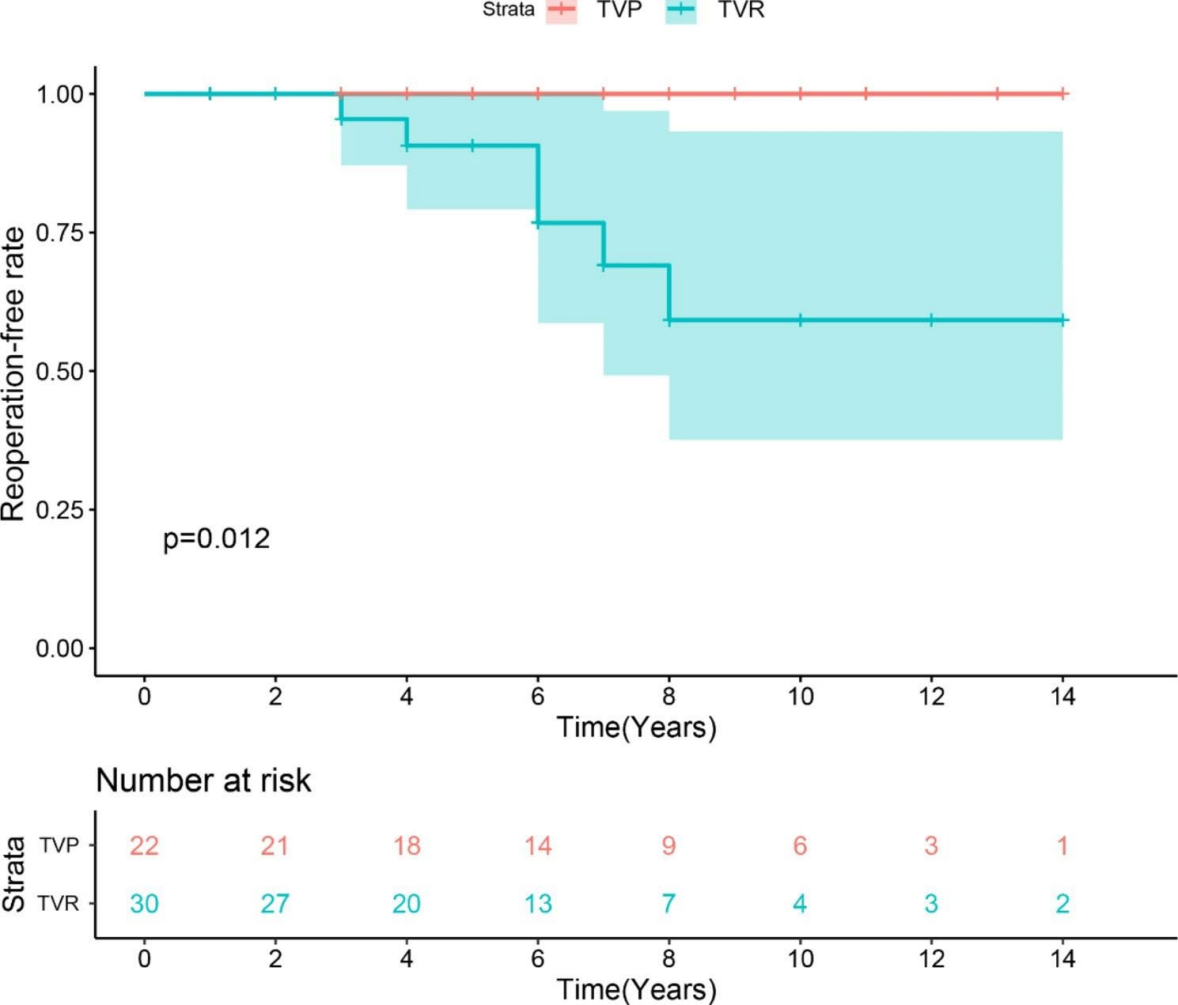
Estimates of free from reoperation rate of patients with TIE who underwent TVP and TVR

Six cases (20%) in the TVR group underwent reoperation; two cases (6.67%) of endocarditis reinfections and four cases (13.33%) of artificial valve dysfunction caused by tricuspid thrombosis. No reoperations were performed in the TVP group. A functional graph was drawn with reoperation as the outcome, and the results showed that the mid- and long-term reoperation-free rates were significantly better in the TVP group than in the TVR group (log-rank *p* = 0.012) (Fig. 2).

## Discussion

With the continuous development of imaging techniques such as echocardiography, the diagnosis of TIE is becoming faster and more accurate. An increasing number of patients with TIE can be diagnosed early and treated promptly; therefore, the proportion of patients with TIE requiring surgical treatment is gradually decreasing. However, in clinical practice, the diagnosis of TIE is often more difficult than that of IE in the left chambers of the heart. Owing to the long preoperative course, the early-stage condition often presents with fever, pulmonary infection, and other nonspecific symptoms. The early misdiagnosis rate of TIE is high. Consequently, some patients had severe infection and cardiac insufficiency when they were diagnosed. In this case, surgical treatment is an important tool for removing the foci of infection, repairing structural damage to the heart, correcting heart failure, and preventing complications [12].

In our study, no significant differences were found in baseline data and preoperative general conditions between the two groups. However, the CPB and ACC times were significantly longer in the TVR group than in the TVP group. We believe that this is mainly because some cases in the TVR group were transferred to the TVR group, as TVP could not be performed during the operation or the effect after shaping was not satisfactory.

The vegetation of TIE is usually large and colonizes the valvular tissue. Infections can easily invade the valvular leaflets, annuli, and adjacent tissues. Through intraoperative exploration, we chose different surgical methods according to specific pathological conditions [13]. In this study, suture valvuloplasty which including Kay suture valvuloplasty and De Vega annuloplasty was performed in ten patients and prosthetic ring annuloplasty was performed in thirteen patients in the TVP group. Compared with suture valvuloplasty, prosthetic ring annuloplasty is suitable for the physiological structure of the diaphragmatic valve region, so that the pulling force caused by the reduction of the valve ring is evenly dispersed throughout the valve ring, which reduces the occurrence of conduction system injury. However, the implantation of an artificial valve ring increases the risk of recurrent IE and thrombosis. During the follow-up, we found seventeen cases of mild or moderate valvular regurgitation in the TVP group and regurgitation in all suture valvuloplasty cases. In cases of prosthetic ring annuloplasty, regurgitation occurred in two cases of Carpentier valvuloplasty and five cases of EdwardsMC3 valvuloplasty. The recurrence rate of tricuspid regurgitation after prosthetic ring annuloplasty was significantly lower than that after suture valvuloplasty.

Studies by Dreyfus et al. [16] have also reported that prosthetic ring annuloplasty is superior to suturing in terms of avoiding tricuspid regurgitation and reoperation. In the TVR group, the development of bilobar mechanical valves and biovalves greatly improved patient prognosis, as reported previously [17]. In China, mechanical valves are recommended for young patients with a good long-term prognosis. In clinical practice, specific valve selection should be based on the actual conditions of patients, including sex, age, life expectancy, compliance, history of drug use, special requirements, and economic conditions. In our study, eleven patients in the TVR group used biological valves and twenty-two patients used bilobar mechanical valves.

In this study, the number of patients with perioperative complications in the TVR group(n = 11) was significantly higher than that in the TVP group (n = 2). The reasons for this may be as follows: (1) CPB and ACC times were longer in the TVR group than in the TVP group. Long CPB time increases the destruction of blood cells, consumption of prothrombin, and influx of inflammatory factors, leading to postoperative coagulation dysfunction and increasing the demand for postoperative blood transfusion. In our study, the amount of postoperative erythrocyte transfusion in the TVR group was significantly higher than that in the TVP group; (2) owing to prolongation of CPB and ACC times during the operation, all organs in the TVR group suffered longer tissue perfusion deficiency and increased ischemia-reperfusion injury, and dysfunction of the liver, lung, kidney, and other organs were more likely to occur after surgery, which affected the recovery of patients post-surgery. Therefore, the postoperative ICU stay time and mechanical ventilation time in the TVR group were significantly longer than those in the TVP group; (3) TVR was routinely performed when TIE was combined with a perivalvular abscess, which spread easily to the surrounding conduction system. Cleaning up the focus of infection and mechanical valve implantation may also compress the surrounding conduction tissue; therefore, an atrioventricular block is more likely to occur in the TVR group [18, 19]. In our study, two cases of third-degree atrioventricular block were noted in the TVR group, which was not significantly different from that in the TVP group. We believe that this was because the chief surgeon at our center had extensive experience in dealing with tricuspid valve disease and avoided some operations that might damage the conduction system.

The early- and medium-term survival rates of the two groups were approximately the same, but the long-term reoperation rate in the TVP group was significantly lower than that in the TVR group (0% vs. 20%, *p* = 0.029). More patients in the TVR group were re-hospitalized because of valvular infection or dysfunction, which is consistent with the results of a previous study [20]. We believe that the main reason for this is that the valve implanted during TVR is in a relatively low-pressure area, and the blood flow velocity in the tricuspid valve area is slower than that in the mitral and aortic valve areas. Especially in patients with mechanical tricuspid valve replacement, this is more likely to lead to valve stenosis, resulting in valve dysfunction [21, 22]. In our study, four cases of artificial valve dysfunction occurred in the TVR group during postoperative follow-up, and all of them were treated with secondary surgery. Therefore, to prevent postoperative thrombosis, if there are no obvious signs of bleeding after TVR, anticoagulant therapy should be administered as soon as possible, blood coagulation function should be monitored, and international normalized ratio should be maintained between 2.5 and 3.5 as much as possible [23–26]. In contrast, the implantation of foreign bodies during the operation was avoided in the TVP group, and this reduced the risk of postoperative IE recurrence, especially for intravenous drug users [27]. Simultaneously, patients in the TVP group did not require long-term anticoagulant therapy and had a reduced possibility of a cardiac block requiring permanent pacemaker implantation. Therefore, TVP has better surgical benefits for individuals such as intravenous drug users or patients at risk of postoperative anticoagulation [28]. While it cannot be conclusively stated that TVP is superior to TVR, but in terms of overall postoperative benefits, the follow-up cost of TVR is significantly higher than that of TVP which may be attributed to the occurrence of complications during follow-up, readmission, or secondary surgery.

During the follow-up, we found that the incidence of moderate or severe tricuspid regurgitation in the TVP group was significantly higher than that in the TVR group (27.27% vs. 0%, *p* = 0.004). However, considering that both TVP and TVR can completely remove the focus of infection and improve postoperative cardiac function, the right ventricular system has a relatively high tolerance to valvular regurgitation and can tolerate residual or recurrent tricuspid regurgitation. At the same time, with the gradual recovery of cardiac function and the decrease in right ventricular volume load, the tricuspid valve function can be partially recovered, and the TVP group shows an overall higher quality of life after surgery. Therefore, TVP remains a preference of most surgeons for patients with TIE who require surgical treatment [16, 29, 30].

## Limitations

First, this was a single-center study instead of a multicenter controlled study. The time span of the cases included in this study was large, and the improvement in treatments and medical devices over the years will inevitably affect the treatment decisions in different time periods. In future studies, we will aim to conduct joint multicenter research to collect case data in a timely, comprehensive, and accurate manner. We will also strengthen our follow-up efforts to obtain more scientific and objective conclusions.

Second, because this was a retrospective study, the choice between valvuloplasty and valve replacement was mainly based on the intraoperative detection of valvular lesions and the experience or judgment of the surgeon. TVR is more likely to resolve this problem in patients with severe valvular disease, which may have some impact on the observed results. This selection bias is an inherent limitation of the retrospective study design. Therefore, to better compare the impact of surgical methods on prognosis, we should expand the sample size as much as possible and include more prospective studies to analyze or control for clinical and surgical details.

## Conclusions

For TIE requiring surgical treatment, both TVP and TVR are safe and effective surgical methods. We found no significant differences in postoperative mortality and early- and medium-term survival between these two methods. However, the ICU stay and mechanical ventilation time with TVP were shorter and the need for postoperative blood transfusion and the risk of postoperative complications were lower. Additionally, TVP is better than TVR in preventing the need for reoperation in the medium and long terms.

## Data Availability

The data that support the findings of this study are available on request from the corresponding author. The data are not publicly available due to privacy or ethical restrictions.

## List of Abbreviations

ACC: Aortic cross-clamp
CPB: Cardiopulmonary bypass
ICU: Intensive care unit
IE: Infective endocarditis
MODS: Multiple organ dysfunction syndrome
TIE: Tricuspid infective endocarditis
TVP: Tricuspid valvuloplasty
TVR: Tricuspid valve replacement

## Declarations

### Statement

This article is a retrospective study, not a clinical trial, so registration with the ICMJE is not required. A reprint has previously been published [Linfeng Xie et al. posted 17 Sep, 2021] [31].
